# Supplementary material for: Newly Diagnosed Type 2 Diabetes Care between Family Physicians, Endocrinologists, and Other Internists in Taiwan: A Retrospective Population-Based Cohort Study
Source: J Pers Med. 2022 Mar 14;12(3):461. doi: 10.3390/jpm12030461 (PMC8955527; doi:10.3390/jpm12030461)
Supplement: Supplementary file 1 [file jpm-12-00461-s001.zip › jpm-supplementary file.pdf]

Article

Newly Diagnosed Type 2 Diabetes Care between Family Physicians, Endocrinologists, and Other Internists in Taiwan: A Retrospective Population-Based Cohort Study

Pei-Lin Chou [1,†], I-Hui Chiang [1,†], Chi-Wei Lin [1,2], His-Hao Wang [2,3,4], Hao-Kuang Wang [2,5], Chi-Hsien Huang [1], Chao-Sung Chang [2,6], Ru-Yi Huang [1,2,*] and Chung-Ying Lin [7,8,9,10,*]

[1] Department of Family and Community Medicine, E-Da Hospital, Kaohsiung 82445, Taiwan; ngilriw@gmail.com (P.-L.C.); ed109959@edah.org.tw (I.-H.C.); ed104283@edah.org.tw (C.-W.L.); ed103520@edah.org.tw (C.-H.H.)
[2] College of Medicine, I-Shou University, Kaohsiung 82445, Taiwan; ed103631@edah.org.tw (H.-H.W.); ed101393@edah.org.tw (H.-K.W.); ed107670@edah.org.tw (C.-S.C.)
[3] Division of Nephrology, Department of Internal Medicine, E-Da Hospital, Kaohsiung 82445, Taiwan
[4] Department of Medical Quality, E-Da Hospital, Kaohsiung 82445, Taiwan
[5] Department of Neurosurgery, E-Da Hospital, Kaohsiung 82445, Taiwan
[6] Committee for Advanced Medical Technology, E-Da Hospital, Kaohsiung 82445, Taiwan
[7] Institute of Allied Health Sciences, College of Medicine, National Cheng Kung University, Tainan 70101, Taiwan
[8] Department of Occupational Therapy, College of Medicine, National Cheng Kung University, Tainan 70101, Taiwan
[9] Biostatistics Consulting Center, National Cheng Kung University Hospital, College of Medicine, National Cheng Kung University, Tainan 70101, Taiwan
[10] Department of Public Health, College of Medicine, National Cheng Kung University, Tainan 70101, Taiwan
* Correspondence: ruyi@mail.harvard.edu (R.-Y.H.); cylin36933@gmail.com (C.-Y.L.)
† These authors contributed equally to this work.

Supplemental file

The International Classification of Diseases for acute complications

Diabetic Ketoacidosis (ICD-9-CM diagnosis codes: 250.1; ICD-10-CM diagnostic codes: E11.65), hyperosmolar hyperglycemic state (HHS, ICD-9-CM diagnosis codes: 250.2 ; ICD-10-CM diagnostic codes: E08.00-E08.01, E09.00-E09.01, E11.00-E11.01, E13.00-E13.01), and

hypoglycemia (ICD-9-CM diagnosis codes: 250.3, 250.8, 251.1, 251.2; ICD-10-CM diagnostic codes: E08.641, E09.641, E11.641, E13.11, E13.641, E11.610, E11.618, E11.620-E11.622, E11.628, E11.630, E11.638, E11.649, E11.65, E11.69, E16.0, E16.1, E16.2.).

The Data Cleaning Process

The data cleaning process was as follows: First, the data collected for each subject was merged according to the patient's individual identification number among different datasets. Subjects who did not meet the study-specific inclusion criteria were removed (e.g. Type 1 Diabetes Mellitus). Missing data were removed (e.g. gender and level of urbanization). Finally, information that was clearly unreasonable (e.g. age 150 years old) were deleted from the data set.

Supplemental Table S1. The Charlson Comorbidities Index

Comorbidities	ICD9-CM	ICD10
Myocardial infarct	410.X、412.X	I21.X,I22.X,I25.2
Congestive heart failure	428.X	I09.9,I11.0, I13.0, I13.2, I25.5, I42.0, I42.5-I42.9, I43.x,
Peripheral vascular disease	443.9、441.X、785.4、V434、384.8	I70.x, I71.x, I73.1, I73.8, I73.9, I77.1, I79.0, I79.2, K55.1,
Cerebrovascular disease	430.X-438.X	G45.x, G46.x, H34.0, I60.x-I69.x

Dementia	290.X	F00.x-F03.x, F05.1, G30.x, G31.1
Chronic lung disease	490.X-496.X、500.X-505.X、506.4	I27.8, I27.9, J40.x-J47.x, J60.x-J67.x,
Connective tissue disease	710.0、710.1、710.4、714.0-714.2、714.81、725.X	I05.x, M06.x, M31.5, M32.x-M34.x, M35.1, M35.3, M36.0
Ulcer	531.X-534.X	K25.x-K28.x
Chronic liver disease	571.2、571.4、571.5、571.6	B18.x, K70.0-K70.3, K70.9, K71.3-K71.5, K71.7, K73.x,
Diabetes	250.0-250.3、250.7	E10.0, E10.l, E10.6, E10.8, E10.9, E11.0, E11.1, E11.6, E11.8, E11.9, E12.0, E12.1, E12.6, E12.8, E12.9, E13.0,
Diabetes with end organ damage	250.4-250.6	E10.2-E10.5, E10.7, E11.2-E11.5, E11.7, E12.2-E12.5,
Hemiplegia	344.1、342.X	G04.1, G11.4, G80.1, G80.2, G81.x, G82.x, G83.0-G83.4,
Moderate or severe kidney disease	582.X、583.0-583.7、585.X-586.X、588.X	I12.0, I13.1, N03.2-N03.7, N05.2-N05.7, N18.x, N19.x,
Tumor, leukemia, lymphoma	140.X-195.X、200.X-208.X	C00.x-C26.x, C30.x-C34.x, C37.x-C41.x, C43.x, C45.x-
Moderate or severe liver disease	572.2-572.8、456.00-456.21	I85.0, I85.9, I86.4, I98.2, K70.4, K71.1, K72.1, K72.9,
Malignant tumor, metastasis	196.0-199.1	C77.x-C80.x
AIDS	042.0-044.9	B20.x-B22.x, B24.x

Supplemental Table S2. Characteristics of the Family Medicine (FM) and Other Cohorts [a]

	Others N=21216	FM N=21216	p-value
Age	54.74±10.68	56.43±10.43	<.001
Gender - no. (%)			>.999
Female	12528(59.05)	12528(59.05)	
Male	8688(40.95)	8688(40.95)	
Urbanization - no. (%)			>.999
High	7704(36.31)	7704(36.31)	
Moderate	9313(43.90)	9313(43.90)	
Low	4199(19.79)	4199(19.79)	
Testing frequency (per year)			
HbA1C	1.16±2.11	3.09±2.73	<.001
Lipid profile	2.79±5.16	7.07±7.03	<.001
ACR	0.39±1.27	0.45±1.13	<.001
Insulin (1-month)[b]	0.48±2.36	0.3±1.46	<.001
Cost[c]	239.59±948.68	490.88±621.38	<.001

Comorbidities - no. (%)			
Myocardial infarct	893(4.21)	785(3.7)	0.0071
Congestive heart failure	2031(9.57)	1689(7.96)	<.001
Peripheral vascular disease	989(4.66)	1083(5.1)	0.0342
Cerebrovascular disease	2939(13.85)	2753(12.98)	0.0081
Dementia	54(0.25)	43(0.2)	0.2635
Chronic lung disease	4360(20.55)	4608(21.72)	0.0032
Connective tissue disease	277(1.31)	254(1.2)	0.3152
Ulcer	6116(28.83)	6044(28.49)	0.4395
Chronic liver disease	5117(24.12)	4683(22.07)	<.001
Hemiplegia	17(0.08)	32(0.15)	0.032
Moderate or severe kidney disease	830(3.91)	698(3.29)	0.0006
Tumor, leukemia, lymphoma	2100(9.9)	2024(9.54)	0.2129
Moderate or severe liver disease	554(2.61)	187(0.88)	<.001
Malignant tumor, metastasis	299(1.41)	301(1.42)	0.9345
AIDS			
Acute Complications - no. (%)	416(1.96)	321(1.51)	<.001

[a]Plus-minus values are means ± SD.

[b]Insulin (1-month) means the initial prescription frequencies during the first month of diagnoses.

[c]Cost means the average diabetes-related annual costs that expressed in U.S. dollars.

ACR = albumin to creatinine ratio. AIDS = acquired immunodeficiency syndrome. HbA1c = glycated hemoglobin.

Supplemental Table S3. Characteristics of Others and Internal Medicine (IM) Cohorts[a]

	Others N=55858	IM N=55858	p-value
Age	55.29±11.13	55.44±11.06	0.0206
Gender - no. (%)			>.999

Female	34451(61.68)	34451(61.68)	
Male	21407(38.32)	21407(38.32)	
Urbanization - no. (%)			>.999
High	23197(41.53)	23197(41.53)	
Moderate	24110(43.16)	24110(43.16)	
Low	8551(15.31)	8551(15.31)	
Testing frequency (per year)			
HbA1C	1.16±2.1	2.61±2.81	<.001
Lipid profile	2.83±5.28	6.66±7.28	<.001
ACR	0.39±1.21	0.71±1.82	<.001
Insulin (1-month)[b]	0.48±2.34	0.49±1.84	0.3043
Cost[c]	251.67±851.56	515.48±941.49	<.001
Comorbidities - no. (%)			
Myocardial infarct	2683(4.8)	2997(5.37)	<.001
Congestive heart failure	6510(11.65)	7412(13.27)	<.001
Peripheral vascular disease	3144(5.63)	2965(5.31)	0.0185
Cerebrovascular disease	9434(16.89)	9539(17.08)	0.4028
Dementia	164(0.29)	165(0.3)	0.956
Chronic lung disease	11994(21.47)	11841(21.2)	0.2638
Connective tissue disease	746(1.34)	649(1.16)	0.009
Ulcer	17916(32.07)	18465(33.06)	0.0005
Chronic liver disease	12928(23.14)	13853(24.8)	<.001
Hemiplegia	53(0.09)	42(0.08)	0.2589
Moderate or severe kidney disease	2581(4.62)	3164(5.66)	<.001
Tumor, leukemia, lymphoma	5416(9.7)	5530(9.9)	0.2513
Moderate or severe liver disease	1550(2.77)	2424(4.34)	<.001
Malignant tumor, metastasis	720(1.29)	721(1.29)	0.9788
AIDS			
Acute Complications - no. (%)	1166(2.09)	1560(2.79)	<.001

[a]Plus-minus values are means ± SD.

[b]Insulin (1-month) means the initial prescription frequencies during the first month of diagnoses.

[c]Cost means the average diabetes-related annual costs that expressed in U.S. dollars.

ACR = albumin to creatinine ratio. AIDS = acquired immunodeficiency syndrome. HbA1c = glycated hemoglobin.

Supplemental Table S4. Characteristics of Others and EndocrinologistCohorts[a]

	Others N=42481	Endocrinologist N=42481	p-value
Age	52.46±11.64	53.16±11.46	<.001
Gender - no. (%)			>.999
Female	25861(60.88)	25861(60.88)	
Male	16620(39.12)	16620(39.12)	
Urbanization - no. (%)			>.999
High	21972(51.72)	21972(51.72)	
Moderate	15694(36.94)	15694(36.94)	
Low	4815(11.33)	4815(11.33)	
Testing frequency (per year)			
HbA1C	1.19±2.15	4.73±3.52	<.001
Lipid profile	2.84±5.41	10.33±8.33	<.001
ACR	0.39±1.22	1.25±1.94	<.001
Insulin (1-month)[b]	0.55±21.29	0.86±2.46	0.0032
Cost[c]	227.58±733.63	922.83±977.41	<.001
Comorbidities - no. (%)			
Myocardial infarct	1550(3.65)	1419(3.34)	0.0144
Congestive heart failure	3654(8.6)	3104(7.31)	<.001
Peripheral vascular disease	1689(3.98)	1745(4.11)	0.3293
Cerebrovascular disease	4955(11.66)	4982(11.73)	0.7732
Dementia	73(0.17)	62(0.15)	0.3434
Chronic lung disease	7288(17.16)	7246(17.06)	0.702
Connective tissue disease	403(0.95)	426(1.00)	0.4221
Ulcer	10925(25.72)	10549(24.83)	0.003
Chronic liver disease	8085(19.03)	7580(17.84)	<.001
Hemiplegia	29(0.07)	27(0.06)	0.7892
Moderate or severe kidney disease	1537(3.62)	1161(2.73)	<.001

Tumor, leukemia, lymphoma	4608(10.85)	4585(10.79)	0.7995
Moderate or severe liver disease	1009(2.38)	579(1.36)	<.001
Malignant tumor, metastasis	852(2.01)	852(2.01)	>.999
AIDS			
Acute Complications - no. (%)	852(2.01)	935(2.2)	0.0472

[a]Plus-minus values are means ± SD.

[b]Insulin (1-month) means the initial prescription frequencies during the first month of diagnoses.

[c]Cost means the average diabetes-related annual costs that expressed in U.S. dollars.

ACR = albumin to creatinine ratio. AIDS = acquired immunodeficiency syndrome. HbA1c = glycated hemoglobin.

Supplemental Table S5. Prediction of the occurrence of acute complications [a]

	FM vs. others		Endocrine vs. others		IM vs. others	
	sHR (95% CI)	p-value	sHR (95% CI)	p-value	sHR(95%CI)	p-value
FM vs. Others	0.69(0.65-0.72)	<.001				
Endocrine vs. Others			0.93(0.91-0.95)	<.001		
IM vs. Others					0.81(0.78-0.83)	<.001
Age	1.01(1.00-1.01)	<.001	1.01(1.01-1.01)	<.001	1.01(1.01-1.01)	<.001
Male vs. Female	1.02(0.97-1.06)	0.441	0.97(0.95-0.99)	0.007	0.99(0.96-1.02)	0.634
Urbanization						
High	REF.		REF.		REF.	
Moderate	1.34(1.27-1.42)	<.001	1.35(1.32-1.38)	<.001	1.28(1.24-1.32)	<.001
Low	1.64(1.55-1.74)	<.001	1.50(1.46-1.54)	<.001	1.42(1.36-1.48)	<.001
Test frequency (per year)						
HbA1C	1.03(1.02-1.04)	<.001	1.00(1.00-1.01)	0.033	1.00(1.00-1.01)	0.319
Lipid profile	1.01(1.01-1.02)	<.001	1.01(1.01-1.01)	<.001	1.01(1.01-1.01)	<.001
ACR	1.06(1.06-1.07)	<.001	1.06(1.05-1.06)	<.001	1.05(1.05-1.06)	<.001

Insulin (1-month)[b]	1.00(1.00-1.00)	0.012	1.01(1.01-1.01)	<.001	1.01(1.01-1.01)	<.001
Cost[c]	1.00(1.00-1.00)	<.001	1.00(1.00-1.00)	<.001	1.00(1.00-1.00)	<.001
Comorbidities						
Myocardial infarct	1.12(1.05-1.19)	0.001	1.00(0.98-1.03)	0.747	1.10(1.06-1.15)	<.001
Congestive heart failure	1.64(1.55-1.72)	<.001	1.54(1.51-1.57)	<.001	1.47(1.42-1.53)	<.001
Peripheral vascular disease	2.31(2.19-2.44)	<.001	2.42(2.36-2.47)	<.001	2.73(2.64-2.83)	<.001
Cerebrovascular disease	1.51(1.44-1.58)	<.001	1.37(1.35-1.40)	<.001	1.34(1.30-1.38)	<.001
Dementia	1.36(1.16-1.60)	<.001	1.28(1.20-1.37)	<.001	1.21(1.08-1.36)	0.002
Chronic lung disease	0.87(0.83-0.91)	<.001	0.87(0.85-0.89)	<.001	0.83(0.80-0.85)	<.001
Connective tissue disease	1.04(0.92-1.19)	0.520	0.96(0.91-1.03)	0.255	0.96(0.88-1.06)	0.442
Ulcer	0.88(0.84-0.92)	<.001	0.82(0.81-0.84)	<.001	0.83(0.81-0.86)	<.001
Chronic liver disease	0.76(0.72-0.81)	<.001	0.82(0.80-0.85)	<.001	0.86(0.82-0.89)	<.001
Hemiplegia	1.09(0.97-1.24)	0.157	0.96(0.92-1.01)	0.127	0.90(0.82-1.00)	0.041
Moderate or severe kidney disease	1.05(0.98-1.14)	0.166	1.24(1.21-1.28)	<.001	1.24(1.18-1.30)	<.001
Tumor, leukemia, lymphoma	0.86(0.79-0.94)	<.001	0.88(0.85-0.91)	<.001	0.94(0.89-0.99)	0.030
Moderate or severe liver disease	1.57(1.27-1.94)	<.001	1.85(1.72-1.99)	<.001	1.42(1.24-1.63)	<.001
Malignant tumor, metastasis	1.37(1.12-1.67)	0.002	1.24(1.12-1.37)	<.001	1.23(1.09-1.39)	<.001
AIDS	NA		NA		NA	

[a]- means not applicable.

[b]Insulin (1-month) means the initial prescription frequencies during the first month of diagnoses.

[c]Cost means the average diabetes-related annual costs that expressed in U.S. dollars.

[d]The subdistribution hazard ratio (sHR) calculated using the Fine and Gray regression hazards model, and p-values were determined using Gray's test.

ACR = albumin to creatinine ratio. AIDS = acquired immunodeficiency syndrome. Endocrine = the endocrinologist cohort. FM = the family medicine cohort. HbA1c = glycated hemoglobin. IM = the other internal medicine cohort.
